# Comparative outcomes of penile skin grafts versus buccal mucosal grafts in urethroplasty for the treatment of extensive anterior urethral strictures

**DOI:** 10.1038/s41598-025-14191-w

**Published:** 2025-08-12

**Authors:** Ahmed Alrefaey, Mohamed Ahmed Anwar, Mostafa Ezzeldeen Abdelmagid, Ibrahim Alaa Eldin Tagrida, Adel Elatreisy, Ahmed Fahim

**Affiliations:** Urology Department, Faculty of Medicine, Al-Azhar university, Cairo, Egypt

**Keywords:** Stricture, Urethra, Buccal mucosa, penile skin, graft, Urethra, Urological manifestations

## Abstract

To compare the outcomes of augmentation urethroplasty (AU) performed with either buccal mucosa graft (BMG) or penile skin graft (PSG) in managing long-segment anterior urethral strictures. A prospective randomized trial involved 98 patients diagnosed with anterior urethral stricture scheduled to AU between June 2022 and December 2024. Participants were randomly assigned to either the PSG or BMG arms. The comparison included patient demographics, clinicopathological characteristics of strictures, and surgical outcomes. The primary outcome was the success rate at 12 months. Secondary outcomes included functional parameters, such as the Urethral Stricture Surgery-Patient Related Outcome Measure (USS-PROM), the International Prostate Symptom Score (IPSS), the International Index of Erectile Function (IIEF) Score, the Male Sexual Health Questionnaire for Ejaculatory Dysfunction (MSHQ-EJD), Q-max, and perioperative complications. The study arms were comparable regarding the preoperative parameters with a mean stricture length ± SD of 6.4 ± 2.3 and 7.9 ± 4.1 cm for PSG and BMG, respectively (*p* = 0.11). After a median follow-up of 20 months (12–30), the success rates of AU with PSG and BMG were comparable (93.2% v/s 97.9%, respectively; *p* = 0.346). There was no statistically significant difference in the IIEF (*p* = 0.8) and MSHQ-EJD (*p* = 0.22). The improvements in USS-PROM: LUTS domain (*p* = 0.19), USS-PROM: peeling voiding score (*p* = 0.62), IPSS (*p* = 0.43), and Q-max (*p* = 0.39) were comparable between the study arms. Clavien–Dindo grade I-III complications were 6.8 and 8.3% (*p* = 0.8), with patients’ satisfaction of 90.9% and 93.8% (*p* = 0.5) for PSG and BMG, respectively. Kaplan–Meier survival analysis showed no statistically significant difference in stricture-free survival among both techniques (hazard ratio 1.19; *p* = 0.275). Our study demonstrates that PSG and BMG techniques for augmentation anterior urethroplasty have high and comparable success rates, with equal patient satisfaction and similarly low morbidity. Neither technique negatively affected sexual or ejaculatory functions.

## Introduction

Augmentation urethroplasty (AU) is conventionally regarded as the most effective approach for addressing non-obliterative long-segment anterior urethral strictures^1^. AU utilizing local skin flaps might yield equivalent results to those of grafts; however, flap outcomes are compromised by complications, including penile torsion, shrinkage, and tissue necrosis^2^.

AU with grafts can be constructed from locally accessible genital skin, with a preference for hairless areas such as the prepuce, distal penile region, and inguinal area. Additionally, grafts may be derived from distant mucosal sites, including buccal, lingual, and saphenous vein. Recently, “tissue-engineered” templates have also been developed for grafting^3^.

Due to its characteristic advantages, buccal mucosa graft (BMG) is the most commonly used graft^4^. However, the availability of BMG may be limited in certain circumstances, including bad oral hygiene, oral leukoplakia, malignant lesions, prior radiotherapy, previous graft harvesting, or longer strictures that may require harvesting BMG from bilateral cheeks. Additionally, potential complications associated with this procedure include donor site scarring, sensory deficits around the perioral region, and impaired jaw mobility^1,5^.

An alternative approach is the utilization of penile skin graft (PSG), which is familiar to the urologists and readily available within the operating field, offering an adequate length for long grafts. This technique can be performed under the same regional anesthesia employed for urethroplasty, thereby mitigating the complications associated with general anesthesia, particularly in patients classified as high-risk^6,7^.

The superiority of BMG over PSG has been established. However, all studies were retrospective, except one prospective randomized trial, which showed no statistically significant difference between the two techniques^1^.

This prospective randomized study aimed to evaluate the outcomes of augmentation urethroplasty performed with either BMG or PSG in managing long-segment anterior urethral strictures.

## Patients and methods

### Study design and ethical details

We designed a prospective, randomized, superiority trial conducted at the Urology Department, Faculty of Medicine, Al-Azhar University in Cairo, Egypt, between June 2022 and December 2024. The local Ethics Committee approved this study (Uro-Surg./MD/2022/0011). The study was registered with ClinicalTrials.gov on 28/9/2023 and has the ID number NCT06056856.

### Patient enrollment and patient allocation

All adult patients (> 18 years) with long segment anterior urethral stricture (> 2 cm) amenable to augmentation urethroplasty were enrolled in the study. We excluded patients with urethrocutaneous fistula, urethral diverticulum, cases of lichen sclerosus, and those with scarred and unsalvageable urethral plates.

The sample size for this study was determined using the G-power software program (G*Power 3.1.9.6 for Mac OS X ) and a priori analysis, with an effect size of 0.5. A statistical power of 80% and a type II statistical error of 20% were used in the calculation. The estimated population was 88, divided randomly into two equal parallel groups utilizing the block randomization method, with a block size of 2 and an allocation ratio of 1:1.

Group I included patients who underwent AU utilizing PSG, and Group II included patients who were managed with AU using BMG.

### Surgical details

In the lithotomy position, a midline perineal incision was performed, followed by an intra-operative evaluation of urethral caliber and the urethral mucosa. For urethroplasty utilizing a PSG, the graft was obtained via a circumferential distal penile incision, with harvesting conducted from the lateral distal penile shaft in either a transverse or longitudinal direction subcoronally. The subepithelial tissue of the graft was thinned to the dermal level and tailored as required^8^. In Group II, the buccal graft harvest procedure commenced with the use of a marking pen to delineate a graft measuring 2.5 cm in width, extending as long as necessary. A local anesthetic, specifically Bupivacaine 0.5% with epinephrine, was injected beneath the graft to ensure optimal analgesia and intraoperative hemostasis. The graft was subsequently incised and dissected from the buccinator muscle, with careful avoidance of Stensen’s duct. The resulting defect was intentionally left open to facilitate closure by secondary intention due to its reduced pain profile. The graft was then pinned and appropriately defatted on the back table and preserved in saline until the time of implantation^9^.

In both groups, access to the pendulous urethra was achieved through penile eversion via the perineal wound. The urethra was preserved from separation from the corporal bodies on one side; it was only mobilized from the midline on the ventral aspect to slightly beyond the midline on the dorsal aspect. The anterior urethra was incised on the dorsolateral aspect. The free graft was then sutured to the opened urethral edge, followed by quilting the graft onto the ventral tunica of the corporal bodies. Finally, the free edges of the graft and the opened urethra were closed over a 14 Fr silicone catheter. A surgical drain was placed if deemed necessary ( Kulkarni’s technique)^10,11^.

### Follow up

Patients received intravenous broad-spectrum antibiotics for 48 to 72 h post-surgery, followed by oral antibiotics until the removal of the catheter. Additionally, they were administered bladder antispasmodics, stool softeners, diazepam to mitigate the risk of erections, and on-demand analgesics. Discharge occurred following drain removal, typically within 2 to 3 days after surgery. All patients were scheduled for routine post-urethroplasty follow-up visits, including an appointment at the urology clinic one week post-surgery for wound assessment and another 30 days post-surgery for catheter removal after peri-catheter retrograde urethrography (RGU). Subsequent follow-up appointments were scheduled three months later to evaluate lower urinary tract symptoms (LUTS), conduct simple uroflowmetry, assess treatment satisfaction, and evaluate sexual function. Thereafter, clinic appointments were extended at three months intervals.

Patients presenting with unfavorable outcomes, those experiencing voiding LUTS, or individuals with a low Q-max of 14 mL/s or less were scheduled for RGU and/or cystoscopy as necessary. Urethroplasty Success was defined as the absence of voiding LUTS and confirmation of sustained urethral patency with RGU after 12 months from surgery, without further interventions, including urethral dilation^4^.

### Patient assessment and measured variables

The pre-operative functional assessment encompassed the International Prostate Symptom Score (IPSS) for evaluating LUTS^12^, Urethral Stricture Surgery - Patient Related Outcome Measure (USS-PROM), which combines the LUTS domain, Peeling’s voiding, and a generic health status domain (EQ-5D-3 L of the EuroQol group)^1^, the International Index of Erectile Function (IIEF) Score^13^, and the Male Sexual Health Questionnaire for Ejaculatory Dysfunction (MSHQ-EJD)^14^.

The anatomic assessment involved uroflowmetry (UFM), RGU, and voiding cystourethrography (VCUG).

### Outcomes

The comparison between the study arms was non-blinded. It included patient demographics, clinicopathological characteristics of urethral strictures, outcomes following urethroplasty, and the incidence of perioperative complications, graded according to the modified Clavien-Dindo classification system^15^. The primary endpoint was the success of the urethroplasty 12 months after the procedure, while secondary endpoints included perioperative complications and functional parameters such as IPSS, IIEF, and MSHQ-EJD.

### Statistics

We employed the Statistical Package for Social Sciences (SPSS Inc., Chicago, IL, version 29.0) in conjunction with G Power software for data analysis. To compare proportions, we used the Chi-square or Fisher exact tests as appropriate. The t-test was utilized to compare the means between the two groups. Each statistical comparison was conducted as “two-sided,” with a significance level of α ≤ 0.05. The Kaplan–Meier method was employed to estimate recurrence-free survival, while the log-rank test was applied to analyze categorical data.

## Results

Ninety-two patients with anterior urethral stricture were included in the study. The stricture etiology was post-inflammatory and iatrogenic in more than 60% of patients, mainly involving the peno-bulbar region.

**Fig. 1:**
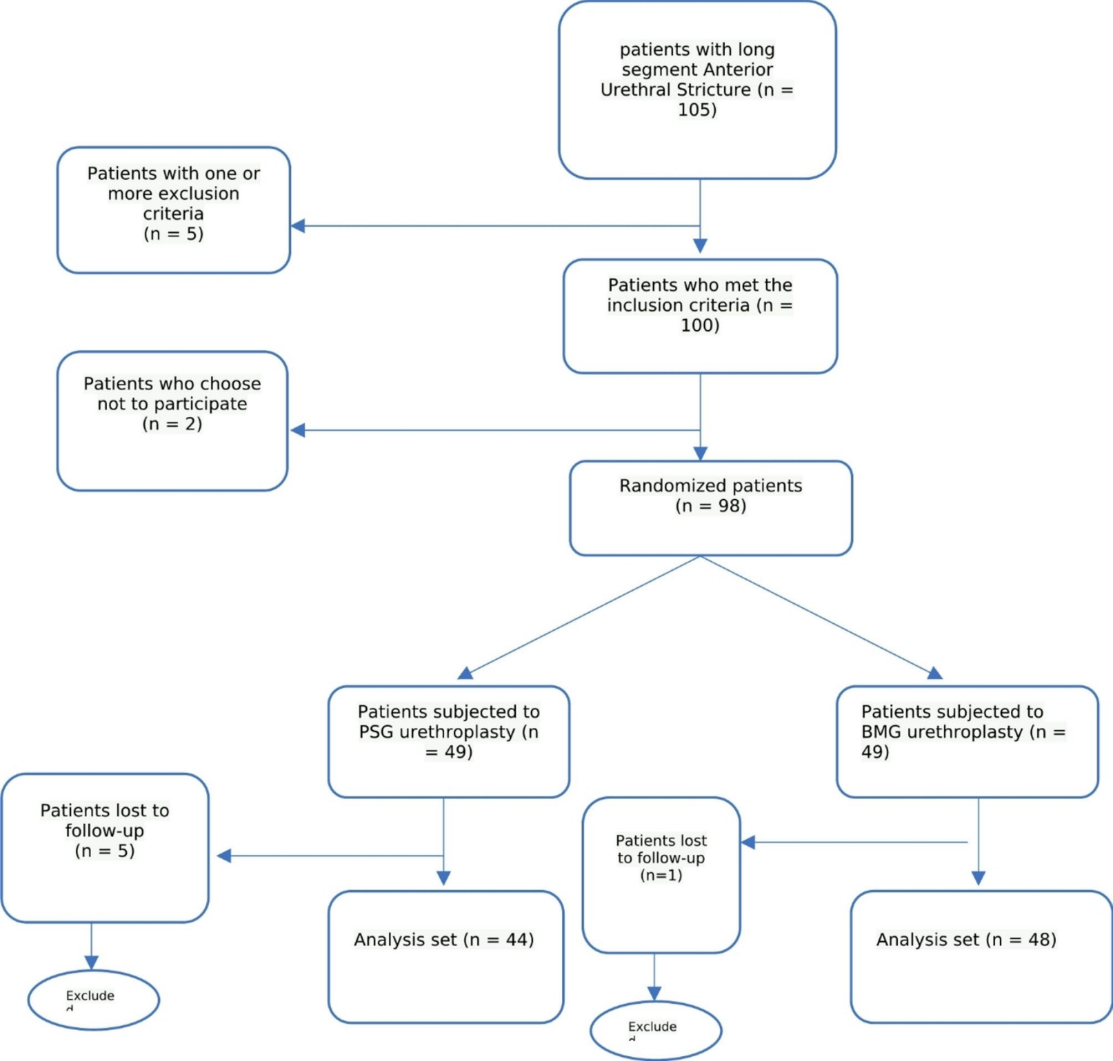
Flow chart of our study population.

44 patients underwent PSG urethroplasty (Group I), and 48 underwent BMG urethroplasty (Group II) (Fig. 1). The study arms were comparable in terms of patient demographics and clinicopathological characteristics, as shown in Table 1.

**Table 1:** Comparison of patient’s demographic, clinico-pathological features of the urethral stricture, and perioperative data among study arms.

	Group I (PSG) (*n* = 44)	Group II (BMG) (*n* = 48)	p-value
Age (years), mean ± SD	40.9 ± 14	40.1 ± 13.8	0.81
BMI (kg/m^2^), mean ± SD	24.4 ± 3.3	23.6 ± 4	0.38
Stricture etiology, N(%)			
Idiopathic	19 (43.18%)	10 (20.83%)	0.008
Inflammatory	10 (22.73%)	17 (35.42%)	
Post-instrumentation	7 (15.91%)	15 (31.25%)	
Traumatic	8 (18.18%)	6 (12.5%)	
Suprapubic catheter, N(%)	8 (18.18%)	9 (18.75%)	0.89
Prior interventions, N(%)	30 (68.18%)	37 (77.1%)	0.46
IIEF grades, N (%)			
No ED	22 (50%)	17 (35.425)	0.14
Mild ED	4 (9.1%)	16 (33.33%)	
Mild to moderate ED	12 (27.27%)	7 (14.58%)	
Moderate ED	3 (6.82%)	5 (10.42%)	
Severe ED	3 (6.82%)	3 (6.25%)	
Stricture location, N(%)			
Proximal bulbar	3 (6.82%)	4 (8.33%)	< 0.001
Distal Bulbar	16 (36.36%)	7 (14.58%)	
Whole bulbar	10 (22.73%)	12 (25%)	
Pan urethral	4 (9.1%)	13 (27.1%)	
Bulbar and proximal penile	11(25%)	3 (6.25%)	
Penile	0	9 (18.75%)	
Stricture length, mean ± SD	6.4 ± 2.3	7.9 ± 4.1	0.11
Lumen obliteration, N (%)			
Partial	33 (75%)	42 (87.5%)	011
Complete	11 (25%)	6 (12.5%)	
Length of harvested graft (cm), mean ± SD	8 ± 2.8	9.6 ± 4.4	0.1
Operative time (min), mean ± SD	115.6 ± 18.9	123.1 ± 23.8	0.15
Hospital Stay (hours), mean ± SD	30.2 ± 17.7	28.3 ± 10.8	0.58
Postoperative complications			
Total	3 (6.82%)	4 (8.3%)	0.8
Wound infection (Grade II)	1 (2.27%)	2 (4.2%)	
Urethro-cutaneous fistula (Grade IIIb)	2 (4.55%)	1 (2.1%)	
Penile curvature (Grade IIIb)	0	1 (2.1%)	

Statistics: X2: Chi-square test, Fisher’s exact test, independent sample T test.

*SD* standard deviation, *BMI* body mass index, *IIEF* international index of erectile function.

We had 11 patients in Group I and six in Group II with complete lumen obliteration and long-segment urethral strictures (> 4 cm), unsuitable for end-to-end anastomosis.

The mean stricture length was 6.4 cm and 7.9 cm, and the graft length was 8 cm and 9.6 cm for Groups I and II, respectively (Table 1). PSG urethroplasty had a slightly shorter operative time compared to BMG urethroplasty, with a mean time of 115 min and 123 min, respectively, and insignificant statistical differences (*p* = 0.15).

No intraoperative complications were reported; however, seven patients (7.6%) experienced postoperative complications of less than Clavien 4, including 3 cases with wound infection, 3 cases with urethral fistula, and one patient with penile chordee. There was no statistically significant difference between the study arms regarding perioperative complications (*p* = 0.8) (Table 1).

At 12 months, there were no statistically significant differences between the study arms regarding the scores of IPSS (*p* = 0.61), USS-PROM: LUTS domain (*p* = 0.19), USS-PROM: peeling voiding domain (*p* = 0.62), USS-PROM: EQ-5D-3 L index (*p* = 0.380), IIEF (*p* = 0.8), erectile function grades (*p* = 0.67), MSHQ-EJD (*P* = 0.22), and Q-max (*p* = 0.39) as depicted in Table 2.

**Table 2:** Comparison of functional and anatomical outcomes between the study arms.

	Group I (PSG) (*n* =44)	Group II (BMG) (*n* =48)	*P*-value
IPSS, mean ± SD			
Preoperative	29.4 ± 6.2	29.8 ± 5.4	0.74
3 months	5.5 ± 5.6	4.3 ± 3.5	0.25
6 months	6.7 ± 7	5.4 ± 4.2	0.32
12 months	6.5 ± 6	5.3 ± 4	0.61
USS-PROM: LUTS domain (mean ± SD)			
Preoperative	16.1 ± 5.2	16.4 ± 5.4	0.81
3 months	6.4 ± 5.1	5.1 ± 3.9	0.23
6 months	6.9 ± 5.2	5.8 ± 3.7	0.21
12 months	6.7 ± 5	5.5 ± 3.2	0.19
USS-PROM: peeling voiding score (mean ± SD)			
Preoperative	3.85 ± 0.61	3.61 ± 0.51	0.25
3 months	1.26 ± 0.52	1.21 ± 0.46	0.45
6 months	1.36 ± 0.72	1.4 ± 0.62	0.76
12 months	1.45 ± 76	1.55 ± 0.89	0.62
USS-PROM: EQ-5D-3 L index score (mean ± SD)			
Preoperative	0.51 ± 0.12	0.55 ± 0.14	0.64
3 months	0.96 ± 0.2	0.94 ± 0.22	0.69
6 months	0.92 ± 0.23	0.9 ± 0.23	0.42
12 months	0.9 ± 0.2	0.89 ± 0.2	0.38
IIEF, mean ± SD			
Preoperative	18.4 ± 5.9	18.5 ± 5.7	0.99
3 months	17.8 ± 6.3	17.7 ± 6.7	0.96
6 months	17.4 ± 6.5	17.1 ± 6.8	0.76
12 months	17.7 ± 6	17.5 ± 6.1	0.8
IIEF grades at 12 months, N (%)			
No ED	19 (43.18%)	19 (39.58%)	0.67
Mild ED	6 (13.64%)	9 (18.75%)	
Mild to moderate ED	12 (27.27%)	12 (25%)	
Moderate ED	6 (13.64%)	4 (8.3%)	
Severe ED	1 (2.27%)	4 (8.3%)	
MSHQ-EJD (mean ± SD)			
Preoperative	10.6 ± 4.5	11.4 ± 4.1	0.5
3 months	10.1 ± 4.1	11.2 ± 4	0.45
6 months	11.3 ± 3.8	12.8 ± 4.1	0.1
12 months	11.5 ± 3.7	12.9 ± 3.9	0.22
QOL grades at 12 months, N (%)			
Delighted	16 (36.4%)	14 (29.2%)	0.52
Pleased	8 (18.2%)	16 (33.3%)	
Mostly satisfied	16 (36.4%)	15 (31.3%)	
Mixed about equally satisfied and dissatisfied	1 (2.3%)	1 (2.1%)	
Mostly dissatisfied	1 (2.3%)	1 (2.1%)	
Unhappy	1 (2.3%)	0 (0%)	
Terrible	1 (2.3%)	1(2.1%)	
Q max (mL/s), mean ± SD			
Preoperative	3.7 ± 3	4.6 ± 3	0.2
3 months	22.3 ± 7.4	22.7 ± 4.2	0.77
6 months	19.1 ± 5.7	20.9 ± 4	0.11
12 months	18.5 ± 6.1	19.5 ± 4.5	0.39
Follow up (months), mean ± SD	21.2 ± 5.3	20.4 ± 5.7	0.473
Urethroplasty success, N (%)	41 (93.2%)	47 (97.9%)	0.346

*SD* standard deviation, *IPSS* international prostate symptom score, *USS-PROM* urethral stricture surgery-patient related outcome measure, *IIEF* international index of erectile function, *MSHQEJD* male sexual health questionnaire for ejaculatory dysfunction, *QOL* quality of life.

Statistics: Chi-square test, Fisher’s exact test, independent sample T test.

The USS-PROM results regarding treatment satisfaction indicated that 40 patients (90.9%) in Group I expressed satisfaction with the surgical outcome, compared to 45 patients (93.8%) in Group II (*P* = 0.5).

The median follow-up duration was 20 Months (interquartile range 12–30 months); this follow-up included the time for recurrence or a minimum of 12 months for patients with no recurrence.

At 12 months, the success rates of urethroplasty with PSG and BMG were high and comparable (93.2% vs. 97.9%, respectively; *p* = 0.346) and remained so throughout the study. (Table 2).

**Fig. 2:**
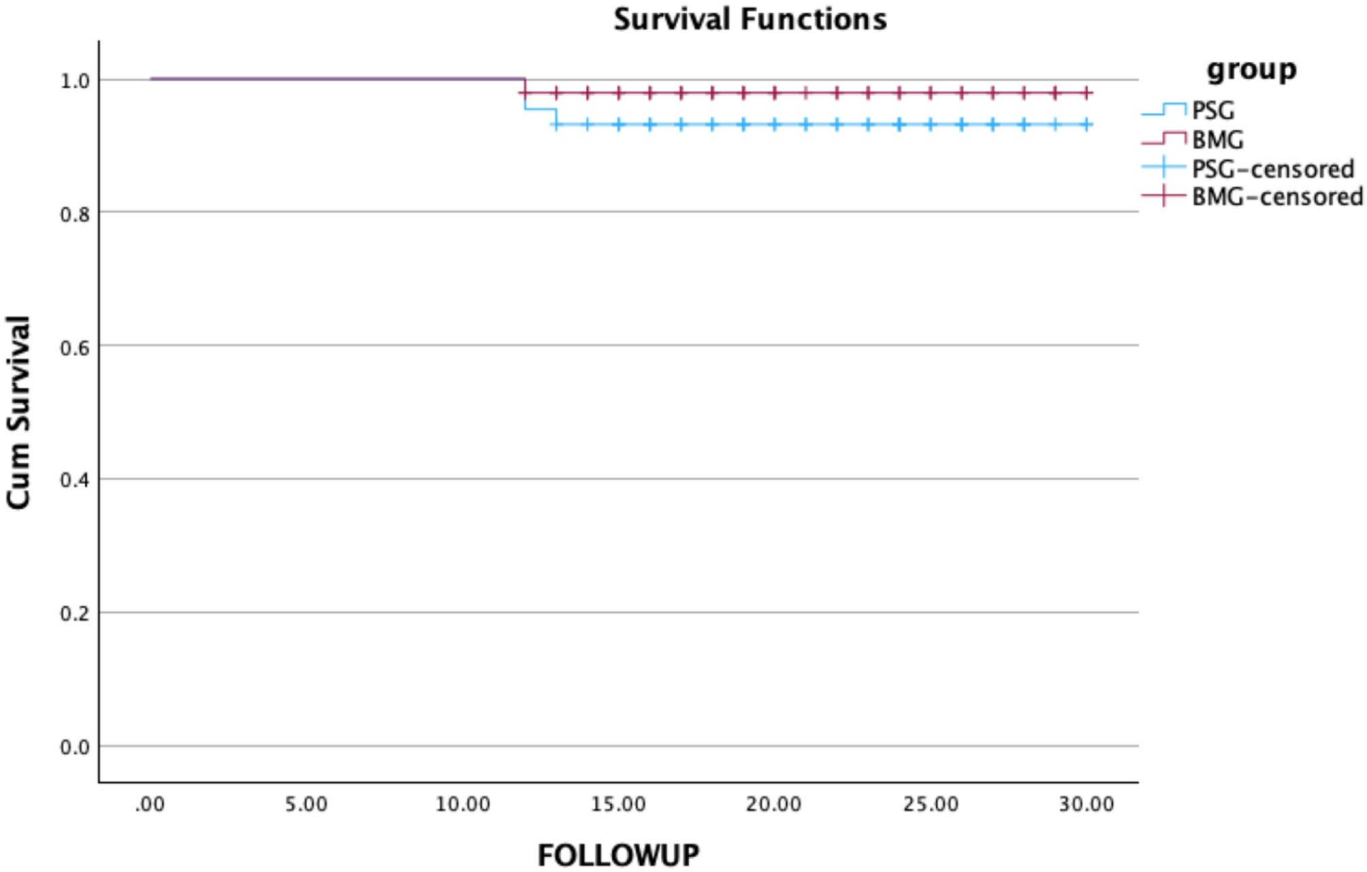
Kaplan–Meier diagram showing comparative recurrence-free survival between the study arms.

Using Kaplan–Meier survival analysis (Fig. 2), we observed no statistically significant difference in stricture-free survival among the study arms (hazard ratio, 1.19; *p* = 0.275).

## Discussion

AU for the treatment of long segment strictures of the anterior urethra, utilizing a BMG in comparison to a PSG, has produced mixed outcomes. In cross-sectional studies, the success rates for BMG have been reported to range between 89% and 96%, while those for PSG have varied from 71 to 86%. The results of non-concurrent cohort studies evaluating the efficacy of AU with BMG and PSG are inconsistent; some studies demonstrate a superior outcome for BMG, whereas others indicate that PSG may be more effective. Concurrent cohort studies have suggested either the superiority of BMG over PSG or comparable efficacy between the two graft types. However, these studies are all non-randomized^1^.

In the current randomized study, we reported a success rate of 93.2% for PSG compared to 97.9% for BMG anterior urethroplasty, with a statistically insignificant difference. These findings are consistent with those of the only prospective randomized trial that compared both surgical techniques conducted by Tyagi and colleagues, who reported success rates of 89% and 91% for PSG and BMG, respectively, with a similarly insignificant p-value (*p* = 0.7)^1^.

The variability in reported success rates following AU may be attributed to several factors. These include differences in the clinicopathological characteristics of the strictures, variations in the surgical techniques, diverse follow-up protocols, distinct study methodologies, and unequal sample sizes^12,16,17^.

Ventral onlay grafts in anterior urethral reconstruction demonstrate the advantage of reduced dissection and improved exposure. Conversely, the dorsal onlay approach is associated with a theoretically favorable graft take. However, both techniques exhibit comparable success rates, ranging from 79 to 90% for ventral onlay grafts and 80–95% for dorsal onlay grafts^4^. In our study, we employed the dorsolateral approach (described by Kulkarni) consistently across all patients in the study arms to ensure uniformity in the surgical approach, as performed by a single expert urethral surgeon (AR).

Augmentation anterior urethroplasty techniques are not likely to inflict any damage or compromise to the neurovascular bundle supplying the erectile tissues. The ejaculatory function may improve because of the alleviation of obstruction; however, it may also deteriorate due to division or traction injuries sustained by the bulbospongiosus muscle during urethroplasty^1^.

The occurrence of sexual dysfunction after anterior urethroplasty has been reported to range from 0 to 40%^18^. Coursey et al. reported that circumcision can be associated with erectile dysfunction (ED) in 27% of cases. Therefore, ED following anterior urethroplasty could be psychogenic rather than organic^19^. Our study showed no significant change in IIEF-ED and MSHQ-EJD scores at 12 months in both study arms compared to pre-operative scores. Also, there were no significant differences regarding erectile and ejaculatory functions in the PSG compared to BMG.

Raber et al.^20^ indicated that the scores on the IIEF-ED remained consistent across both groups in their study. However, they observed a significant improvement in the IIEF-OD scores using PSG compared to BMG. The study did not provide a convincing explanation for this finding. It is worth noting that the research comprised a limited sample size of 30 patients, of whom 17 underwent PSG and 13 underwent BMG.

Patient satisfaction following urethroplasty necessitates both an enhancement in urinary flow and the preservation of sexual function^21^. We have incorporated functional parameters into our study to evaluate postoperative outcomes comprehensively. We noted a significant improvement in LUTS after anterior urethroplasty, evidenced by a reduction in IPSS across both study groups with no reported discomfort or complications at the donor site. Furthermore, we observed that the improvements in all domains of USS-PROM were comparable between the study arms. Our findings align with those documented in existing literature^1^.

Numerous complications associated with urethroplasty have been documented in the literature, such as wound infections, bleeding, penile chordee, and post-void dribbling^22^. In our findings, we reported a rate of postoperative complications at 7.6%, which included wound infections, urethro-cutaneous fistulas, and penile chordee. The PSG and BMG urethroplasty techniques demonstrated a comparable incidence of low-grade postoperative complications, categorized as Clavien grade less than 4.

In sumary, we present a prospective randomized study comparing AU with PSG and BMG in managing long-segment strictures of anterior urethra. Both techniques have comparable outcomes regarding voiding and sexual functions, with high patient satisfaction and success rates of more than 93%. However, the study is subject to limitations, including a relatively short follow-up period of 12 months, with a mean follow-up duration of 20.8 months. Furthermore, objective assessments of post-urethroplasty through urethrocystoscopy were not conducted for all participants and were explicitly reserved for those exhibiting symptoms based on subjective evaluations.

## Conclusion

Our study shows that PSG and BMG techniques for augmentation anterior urethroplasty have high and comparable success rates, with equal patient satisfaction and similarly low morbidity. Neither technique negatively affected sexual or ejaculatory functions.

## Data Availability

Data Availability: Available from the corresponding author on a reasonable request.

## References

[1] -Tyagi, S. et al. Pee’BuSt trial: A single-centre prospective randomized study comparing functional and anatomic outcomes after augmentation urethroplasty with penile skin graft versus buccal mucosa graft for anterior urethral stricture disease. *World J. Urol.* **1**, 1–7 (2022). 10.1007/s00345-021-03843-x PMC8520101 34655304

[2] Dubey, D. et al. Dorsal onlay buccal mucosa versus penile skin flap urethroplasty for anterior urethral strictures: results from a randomized prospective trial. *J. Urol.* **178**, 2466–2469 (2007). 17937943 10.1016/j.juro.2007.08.010

[3] Mangera, A. & Chapple, C. Management of anterior urethral stricture: an evidence-based approach. *Curr. Opin. Urol.* ;**20**:453–458 . 10.1097/MOU.0b013e32833ee8d5 20827208

[4] Shalkamy, O., Elatreisy, A. & Salih, E. Erectile and voiding function outcomes after buccal mucosa graft urethroplasty for long-segment bulbar urethral stricture: ventral versus dorsal onlay technique. *World J. Urol.* **41**, 205–210 (2023). 36460798 10.1007/s00345-022-04220-y

[5] Sharma, G., Sharma, S. & Parmar, K. Buccal mucosa or penile skin for substitution urethroplasty: A systematic review and metaanalysis. *Indian J. Urol.* **36**, 81–88 (2020). 32549657 10.4103/iju.IJU_298_19 PMC7279095

[6] Alsikafi, N. F., Eisenberg, M. & McAninch, J. W. Longterm outcomes of penile skin graft versus buccal mucosal graft for substitution urethroplasty of the anterior urethra. *J. Urol.* **173**, 87–88 (2005).

[7] Lumen, N., Oosterlinck, W. & Hoebeke, P. Urethral reconstruction using buccal mucosa or penile skin grafts: systematic review and meta-analysis. *Urol. Int.* **89**, 387–394 (2012). 22889835 10.1159/000341138

[8] Hudak, S. J., Hudson, T. C. & Morey, A. F. Minipatch’ penile skin graft urethroplasty in the era of buccal mucosal grafting. *Arab. J. Urol.* **10**, 378–381 (2012). 26558053 10.1016/j.aju.2012.03.007 PMC4442938

[9] Zimmerman, W. B. & Santucci, R. A. Buccal mucosa urethroplasty for adult urethral strictures. *Indian J. Urol.* **27**, 364–370 (2011). 22022061 10.4103/0970-1591.85441 PMC3193738

[10] Kartal, I. et al. Comparison between dorsal onlay and one-sided dorsolateral onlay buccal mucosal graft urethroplasty in long anterior urethral strictures. *IJU* **27**, 719–724 (2020). 10.1111/iju.14286 32533574

[11] Islam Mf, Haque, M. et al. Dorsolateral onlay OMG urethroplasty through unilateral urethral mobilization in anterior urethral Stricture - Our experience in Dhaka medical college hospital and Salam urology & transplantation foundation of Bangladesh (SUTF). *J. Urol.* **14**, 22–25 (2011).

[12] Barbagli, G., Guazzoni, G. & Lazzeri, M. One- stage bulbar urethroplasty: retrospective analysis of the results in 375 patients. *Eur. Urol.* **53**, 828–833 (2008). 18243497 10.1016/j.eururo.2008.01.041

[13] Rosen, R. C. et al. Development and evaluation of an abridged, 5-item version of the international index of erectile function (IIEF-5) as a diagnostictool for erectile dysfunction. *Int. J. Impot. Res.* **11**, 319–326 (1999). 10637462 10.1038/sj.ijir.3900472

[14] Rosen, R. C. et al. *Development and Validation of four-item Version of Male Sexual Health Questionnaire To Assess Ejaculatory Dysfunction*69805 (Urology, 2007). 10.1016/j.urology.2007.02.036 17482908

[15] Dindo, D., Demartines, N. & Clavien, P. A. Classification of surgical complications: a new proposal with evaluation in a cohort of 6336 patients and results of a survey. *Ann. Surg.* **240**, 205–213 (2004). 15273542 10.1097/01.sla.0000133083.54934.ae PMC1360123

[16] Lumen, N. et al. Ventral onlay graft urethroplasty using genital skin or buccal mucosa in the treatment of bulbar strictures: a retrospective analysis of 41 cases. *Curr. Urol.* **2**, 10–15 (2008).

[17] D’hulst, P. et al. Patient-reported outcomes after buccal mucosal graft urethroplasty for bulbar urethral strictures: results of a prospective single-centre cohort study. *BJU Int.* **126**, 684–693 (2020). 32512634 10.1111/bju.15131

[18] Shalkamy, O. et al. Erectile function after different techniques of bulbar urethroplasty: does urethral transection make a difference? *BMC Urol.* **23**, 140 (2023). 37620812 10.1186/s12894-023-01281-y PMC10463440

[19] Coursey, J. W. et al. Erectile function after anterior urethroplasty. *J. Urol.* **166**, 2273–2276 (2001). 11696750

[20] Raber, M. et al. Dorsal onlay graft urethroplasty using penile skin or buccal mucosa for repair of bulbar urethral stricture: results of a prospective single center study. *Eur. Urol.* **48**, 1013–1017 (1005). 10.1016/j.eururo.2005.05.003 15970374

[21] Bertrand, L. A. et al. Measuring and predicting patient dissatisfaction after anterior urethroplasty using patient reported outcomes measures. *J. Urol.* **196**, 453–461 (2016). 26907509 10.1016/j.juro.2016.01.117 PMC4969128

[22] Hussein, M. M. et al. Urethroplasty for treatment of long anterior urethral stricture: buccal mucosa graft versus penile skin graft-does the stricture length matter? *Int. Urol. Nephrol.* **48**, 1831–1835 (2016). 27401984 10.1007/s11255-016-1366-0

